# Gamma radiation shielding characteristics of various spinel ferrite nanocrystals: a combined experimental and theoretical investigation

**DOI:** 10.1039/d0ra08372k

**Published:** 2021-02-19

**Authors:** Rajkumar M. Lokhande, Vithal Vinayak, Sachin V. Mukhamale, Pankaj P. Khirade

**Affiliations:** Department of Physics, Shirish Madhukarrao Chaudhari College, Jalgaon MS 425001 India rajml@gmail.com; Department of Chemistry, Shri Chhatrapati Shivaji College Omerga Osmanabad MS 413606 India; Department of Physics, Shri Pundlik Maharaj Mahavidyalaya Nandura Rly MS 443404 India; Department of Physics, Shri Shivaji Science College, Amravati MS 444603 India pankajkhirade@gmail.com

## Abstract

This work presents the facile synthesis of Ni, Mn, Zn, Cu and Co spinel ferrite nanocrystals *via* sol–gel auto-ignition and the investigation of their structural and gamma ray shielding characteristics. Experimentally, gamma ray shielding parameters are determined with different gamma ray sources and NaI(Tl) scintillation detector and theoretically *via* Monte-Carlo simulation (Geant4) as well as NIST-XCOM database. X-ray diffractograms elucidate the cubic spinel structure without any contaminating phases for all synthesized nano-ferrites. TEM results evidence the formation of ultrafine crystallites in nano-regime dimensions. Nanocrystalline spinel ferrites in pellet form have been exposed to gamma radiation from diverse sources by changing the radiation dose intensity. The comparative study of the linear attenuation coefficient, mass attenuation coefficient, total atomic cross section, total electronic cross section, effective atomic number, effective electron density and half value layer for manufactured spinel ferrites is carried out using NIST-XCOM and Geant4 at 122–1330 keV. Gamma ray energy absorption buildup factor (EABF) is investigated for five selected ferrites at 100 keV to 1500 keV incident photon energy and penetration depth from 1 to 40 mfp using geometric progression (G-P) fitting technique. EABF is found to be maximum at an intermediate region, mainly attributed to the Compton scattering process. Zinc ferrite exhibits a higher value of EABF among other ferrites, which mainly depends on the chemical composition of the material and crystallite size effect. The EABF is investigated as a function of penetration depth and is found to be maximum for a penetration depth of 40 mfp. Experimental and theoretical simulation results are found to be in good agreement. The Monte-Carlo simulation of radiation interaction with materials has evidenced to be an excellent approximation tool in exploring spinel ferrite performance in radiation atmosphere.

## Abstract 2

A detailed description of the structural and gamma ray shielding parameters of various spinel ferrite nanocrystals.

## Graphical abstract

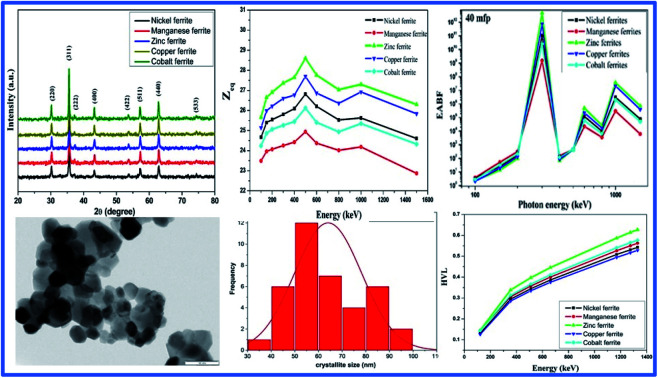

## 1. Introduction

Nanotechnology is one of the fastest growing scientific fields with applications in many diverse areas, including electronics. The term nanoelectronics refers to the use of nanotechnology in electronic components with critical dimensions and size ranging between 1 nm and 100 nm.^1^ All electronics devices manufactured by humans are continuously exposed to diverse types of radiation from natural sources as well as man-made sources. The miniaturization of electronic devices and growing integration of electronic components may have an undesirable impact on the component sensitivity towards ionizing radiations.^2^

Currently, spinel ferrites of nano-dimensions are technologically trending materials due to their distinct electromagnetic characteristics.^3–5^ Spinel ferrites (M–Fe_2_O_4_) have several applications such as in transformers, inductors, capacitors, isolators, circulators, gyrators, phase shifters, reconfigurable antennas, spintronics memory devices, wireless mobile communication, and biomedical instrumentation.^6–10^

Gamma rays are a kind of electromagnetic radiation, and are the packets of energy called photons (*hv*) emitted by the nucleus of some radionuclides resulting in radioactive decay. Gamma rays are most energetic photons in the electromagnetic spectrum having energies beyond 100 keV.^11^ Gamma radiation interacts with matter through ionization through three phenomena: photoelectric effect, Compton scattering and pair production. The enormously high energy of gamma rays permits them to enter just about everything.^12^ They can even pass-through skin, teeth, and bones and destroy living cells, produce gene mutations which cause cancer. This makes gamma rays extremely hazardous.^13^ Gamma radiation is powerful and can affect most electrical components. Simple equipment including motors, switches, incandescent lights, wiring, and solenoid are fairly radiation resistant and might never show any radiation impacts, even after exposure to large radiation. However, diodes and computer chips are much more sensitive to gamma radiation. Diodes and computer chips will show very slight functional damage up to about 50 to 100 Sv.^14,15^

The effect of gamma radiation on electronic memory devices has been investigated by I. Fetahović *et al.*^16^ The impact of direct ionizing radiation on semiconductor memory performance has been scrutinized by using Monte-Carlo simulation technique. The internal crystalline structure of the materials of electronic components is disrupted on interaction with gamma radiation. The functioning of electronic components breaks down and then fails when they are exposed to significant gamma radiation.

Ionizing radiation creates hole–electron couples in the electronic components, changes the transistor parameters and eventually destroys them. It can also cause leakage currents among circuits. As radiation particles voyage through a material they transfer part of their energies to the electrons and the nucleus of the material and rupture the chemical bonds, producing ionization and atomic displacement.^17^ For example, proton damage to a transistor consists of ionization and results in damage.^18^

In order to protect the electronic devices from gamma radiation several approaches are being established. Likewise, some electronics can be toughened so that they are not influenced as much by larger gamma radiation doses by providing shielding or by coating with radiation-resistant materials.^19^ Most nuclear reactors and electronic sectors can only maintain their functionality in radioactive environments with tungsten or lead shielding.^20^ It is heavy and bulky, and its design, installation and replacement are complex and expensive processes. The mass attenuation coefficient, total atomic cross section, total electronic cross section, energy absorption buildup factor (EABF) and exposer buildup factor (EBF) are the basic parameters for measuring the interaction of radiation with matter, which can be applied for the shielding purposes.^21^

In the literature, the comparison of the experimental results on nanomaterials, soils, glass materials, composite materials, different detector materials, rocks and concrete, polymer, and biomaterials with the NIST-XCOM (photon cross sections database) and Monte-Carlo simulations is available.^22,23^

R. H. Kadam *et al.* have determined the mass attenuation coefficient of magnesium ferrite produced *via* ceramic method.^24^ The Hubbel's mixture rule is utilized to find the mass absorption coefficient and related parameters for the prepared MgFe_2_O_4_.^25^ S. D. Raut *et al.* have investigated the gamma ray energy absorption and exposure buildup aspects of Co/Zn/Ni/Mg spinel ferrites utilizing G-P fitting method in the energy range of 0.015–15.00 MeV up to the penetration depth of 40 mfp.^26^

However, there are no noteworthy literature available on the shielding characteristics of spinel ferrite electronic materials. Monte-Carlo simulation is found to be the most effective tool to determine the radiation interaction parameters in diverse kinds of materials, compounds and composites for shielding properties. Demonstrating the photon attenuation properties of materials *via* computer software offers superior accuracy and flexibility of use than the experimental procedure. There are many Monte-Carlo simulation codes available for the study of radiation transport, particle physics, medical physics, cosmo-physics, radiotherapy, and radiation biology, namely, MCNP, GEANT4 and FLUKA.^27–29^ Geant4 is a Monte-Carlo simulation code based on C++, an object-oriented programming method, and is freely available. Geant4 code is applicable for measurement of approximation of photon mass attenuation coefficients for different types of most common scintillation crystal detectors at different energies.^30^

The aim of this research work is to fabricate single-phase nanocrystalline spinel ferrite materials *via* the ecofriendly sol–gel technique and to inspect the performance of these materials when exposed to gamma radiation in order to establish their functionality in a radiation atmosphere. The experimental procedure has been used to test the radiation attenuation parameters of spinel ferrites. Diverse kinds of spinel ferrites *viz.* nickel ferrite (NiFe_2_O_4_), manganese ferrite (MnFe_2_O_4_), zinc ferrite (ZnFe_2_O_4_), copper ferrite (CuFe_2_O_4_) and cobalt ferrite (CoFe_2_O_4_) have been exposed to ionizing gamma radiation by varying the radiation dose intensity. The impact of direct ionizing radiation on spinel ferrite materials' shielding characteristics has been explored by using Monte-Carlo simulation (Geant4) and NIST-XCOM database. Also, a theoretical study has been undertaken to get information on the energy absorption buildup factor of five ferrite materials using G-P fitting method at incident photon energy of 100 keV to 1500 keV up to penetration depths of 40 mfp. The obtained radiological data can be very much useful for elucidating the gamma ray shielding characteristics of spinel ferrite electronic materials.

## 2. Experimental and calculation method

### 2.1 Synthesis of nanocrystalline spinel ferrite

#### Materials

Analytical reagent (AR) grade nickel nitrate (Ni(NO_3_)_2_·6H_2_O), manganese nitrate (Mn(NO_3_)_2_·6H_2_O), zinc nitrate (Zn(NO_3_)_2_·6H_2_O), copper nitrate (Cu(NO_3_)_2_·6H_2_O), cobalt nitrate (Co(NO_3_)_2_·6H_2_O), ferric nitrate (Fe(NO_3_)_3_·9H_2_O) and l-ascorbic acid (C_6_H_8_O_6_) used as precursors with 99.9% purity were procured from Sigma-Aldrich and used as received without further distillation.

### 2.2 Synthesis and characterizations

Nanocrystalline spinel ferrite materials *viz.* nickel ferrite (NiFe_2_O_4_), manganese ferrite (MnFe_2_O_4_), zinc ferrite (ZnFe_2_O_4_), copper ferrite (CuFe_2_O_4_) and cobalt ferrite (CoFe_2_O_4_), were efficiently produced *via* the sol–gel auto-ignition technique and l-ascorbic acid as a combustion agent. The AR grade nitrates of Ni, Mn, Zn, Cu, Co and l-ascorbic acid were liquified in distilled water distinctly to accomplish a homogenous solution. The precursor metal nitrates were dissolved together with the least amount of distilled water essential to achieve a clear solution. The reaction procedure was done in an air atmosphere deprived of the shield of inert gases. The metal nitrates to combustion agent (l-ascorbic acid) ratio was preserved as 1 : 3. Liquified ammonia was added dropwise to maintain the pH at the neutral value 7. The auto-ignition reaction was carried out for few hours with continuous thermal treatment at 100 °C to produce the required product. The as-prepared powders were sintered at 750 °C for 6 h and utilized for further examinations. The sintered powder samples were assorted with polyvinyl alcohol (PVA) mediator as a binder and hard-pressed into compact cylindrical pellets with dimensions of 10 mm diameter and less than 3 mm thickness by the isostatic pressing method under a pressure of 550 kg cm^−2^. The pellets were heat-treated in a furnace at 430 °C for 2 h for the exclusion of PVA and then used for radiation exposure.

Xray diffraction (XRD) analysis was conducted on a PANalytical X'pert pro-diffractometer. The wavelength of the X-ray was 1.542 Å (Cu-Kα radiation, 40 kV and 100 mA), in the 2*θ* scale of 20°–80° and at the scanning rate of 0.02°. The bright-field surface topography of the samples and particle size distribution were obtained by transmission electron microscopy (TEM, CM-200 FEG PHILIPS).

### 2.3 Experimental procedures

The synthesized nanocrystalline spinel ferrites in pellet form were irradiated with gamma rays generated by the radioactive sources, ^57^Co, ^133^Ba, ^22^Na, ^137^Cs, ^54^Mn and ^60^Co, *via* the narrow beam geometry setup NaI (Tl) scintillation detector. All these radioactive sources acquired from Bhabha Atomic Research Centre (BARC), Mumbai, India producing different energies *viz.*^57^Co (122 keV), ^133^Ba (356 keV), ^22^Na (511 and 1275 keV), ^137^Cs (662 keV), ^54^Mn (835 keV) and ^60^Co (1173 and 1332 keV) were collimated. The angle between a radioactive source and the pellet materials was kept as 90° for narrow beam analysis. The NaI scintillation detector with narrow beam geometry was utilized as the gamma ray spectroscopic system. The analysis of the amplified signals by selecting a spectrometry system included a (2′′ × 2′′) NaI (Tl) crystal with an energy resolution of 8.2% at 662 keV from the gamma decay of ^137^Cs. The peak measurement energy *versus* count depends on full width at half maxima (FWHM) and the 8 K multichannel analyzer. The diameters of the pellets played an important role in the narrow beam system (source–sample–detector) and were determined using a traveling microscope for more accuracy. The samples were put in a lead-shielded narrow beam geometry set up (source–sample–detector). The sample thickness was selected in order to satisfy the following ideal condition.^31^
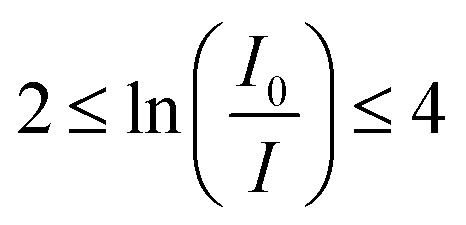


There are two steps in determining the attenuation coefficient by experiment; first to calculate with the source and without the sample and then with the source and with the sample. The present experiments were carried out in an air-conditioned room to avoid possible shifts of the photo-peaks. The room temperature was maintained at 20 ± 1 °C throughout the experiment.

#### Determination of mass attenuation coefficient

According to Beer–Lambert law a parallel ray of X-ray or Gamma ray photons passing through matter is attenuated due to an electromagnetic region (absorption and scattering) and mathematically expressed as:^32^

1 *I* = *I*_0_ exp(−*μ*_m_*t*)

where *I*_0_ and *I* are the incident and transmitted photon intensities of gamma radiations, *μ*_m_ is the mass attenuation coefficient of the nanocrystalline spinel ferrites and *t* is the thickness of the sample. Then mixture rule is applied for a compound or mixture of elements.

2 *μ*_m_ = ∑*iw*_*i*_(*μ*_m_)_*i*_

where *w*_*i*_ and (*μ*_m_)_*i*_ are the weight fraction and mass attenuation coefficient of the *i*^th^ constituent of the ferrites, respectively. The weight fraction (*w*_*i*_) of different chemical compositions is given by3
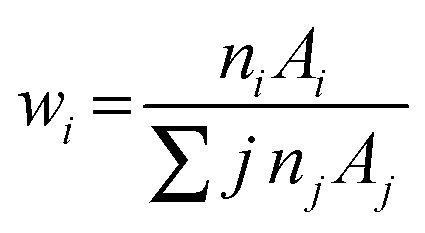


#### Determination of total atomic and electronic cross section

The total atomic cross section (*σ*_t,a_) can be written mathematically as,^33^4
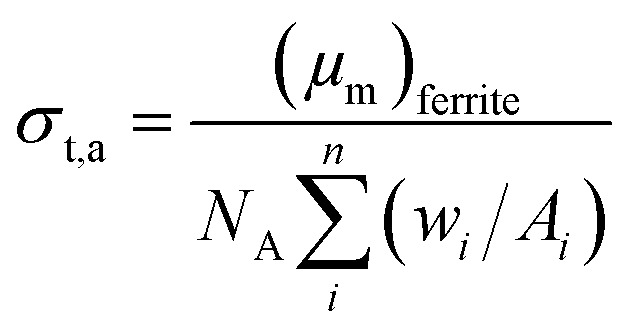
where *N*_A_ is the Avogadro's number and *A*_*i*_ is the atomic weight of the *i*^th^ constituent element of the spinel ferrite. Likewise, the total electronic cross section (*σ*_t,el_) is mathematically expressed as,5
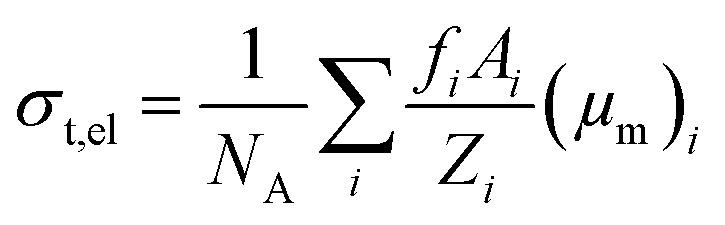
where *f*_*i*_ is the number of atoms of the *i*^th^ constituent element relative to the total number of atoms of the spinel ferrites and *Z*_*i*_ is the atomic number of the *i*^th^ element in the material.

#### Effective atomic number (*Z*_eff_)

The effective atomic number can be estimated using the equation6
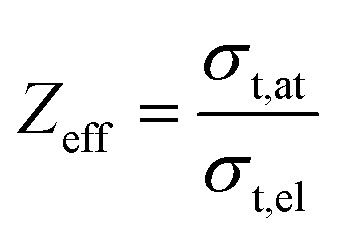


#### Effective electron density (*N*_eff_)



7

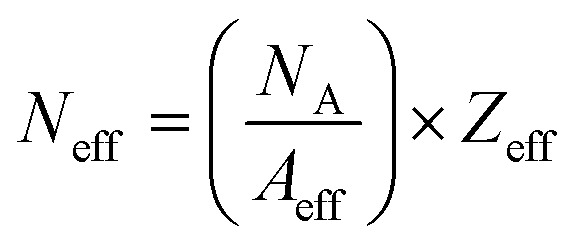

where *A*_eff_ is the effective atomic mass also recognized as the ratio of atomic weight and total number of atoms.

#### Half value layer (HVL)

The half value layer is mathematically expressed as^34^8
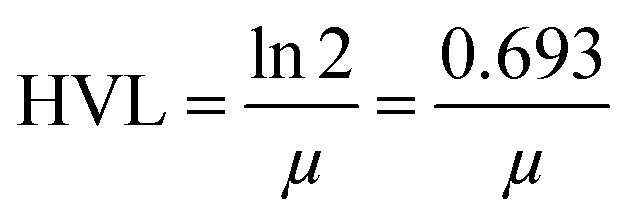


Here, *μ* is the linear attenuation coefficient. The *μ* measured experimentally from the narrow beam geometry setup of NaI(Tl) detector results from the energy and counts observed on the monitor of the system.

#### Determination of energy absorption buildup factor (EABF)

Several researchers studied the energy absorption and exposure buildup factor (EABF) with the help of the G-P fitting technique.^35^ Harima *et al.* developed the G-P fitting technique with the aid of NIST datasheets and ANSI-6.4.3 and illustrated the energy absorption and exposure buildup factor up to the penetration depth of 40 mfp at 0.015 to 15 MeV incident photon energy.^36^ There are three key stages in the determination of the EABF: (a) the determination of equivalence atomic number (*Z*_eq_), (b) the determination of G-P fitting parameters and (c) the computation of EABF. The value of *Z*_eq_ depends on the NIST-XCOM database, partial attenuation coefficient (*μ*_m_)_comp_ and total attenuation coefficient (*μ*_m_)_total_. Statistically, *Z*_eq_ is written in the following form:^37^9
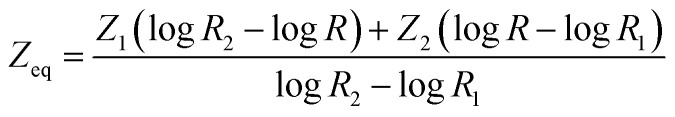


The G-P fitting method is provided by the American National Standards (ANSI/ANS-6.4.3-1991).^38^ Using the interpolation formula, five G-P fitting parameters (*a*, *b*, *c*, *d* and *X*_k_) for the selected samples were computed at different incident photon energies (100–1500 keV) using equivalent atomic number (*Z*_eq_) up to the penetration depth of 40 mfp. The interpolated values were obtained by using the following equation:^39^10
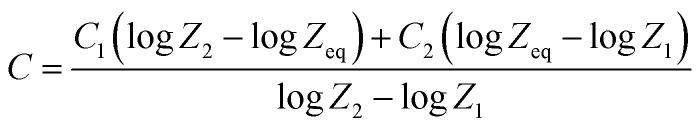


The calculation of the energy absorption buildup factor with the assistance of the G-P fitting formula depends on the shielding thickness as given by following equations:
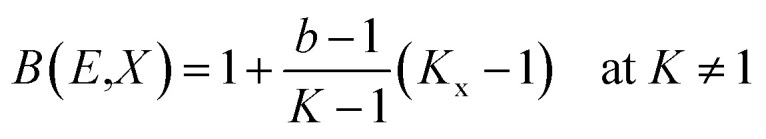
*B*(*E*,*X*) = 1 + (*b* − 1)** **at *K* = 111
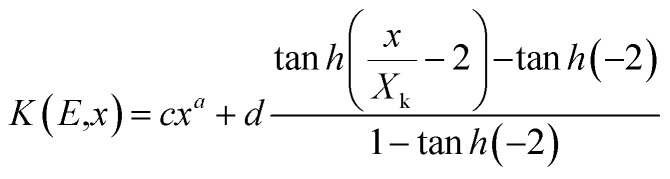
where *b* is the buildup factor at 1 mfp and *K* is a multiplication factor for dose through 1 mfp photon penetration.

#### Monte-Carlo simulation

Geant4 is a platform for the simulation of the passage of particles through matter utilizing Monte Carlo methods. Geant4 is an object-oriented toolkit contingent on the C++ programming language, which can be used for the measurement of radiation interaction with matter at a wide energy range of 250 eV to 100 TeV. The study of electromagnetic radiation in the Geant4 environment is available for low electromagnetic (EM) test package; we studied the EM test 13, 15 and 18 for low EM interaction. The Geant4 method for electromagnetic package was especially applied for narrow beam geometry. The study was carried out in the UNIX operating system Geant4 version 9.06.p01 for all gamma sources. The attenuation coefficient was calculated using computer environment and Geant4 application G4RunManager, the observed result was due to photo-electric effect, Compton scattering and pair production phenomenon. The primary information is required for the construction of the detector geometry of the electromagnetic package for Geant4 simulation. There are three stages; in the first stage of construction, the narrow beam geometry set up of a monochromatic source exposes the selected gamma radiation to the material and detector and a unique distance between source–sample–detector is set. The second stage is the setting of the energy, here we have selected the energy regions of 122 keV, 356 keV, 511 keV, 662 keV, 1170 keV, 1275 keV and 1330 keV, chemical composition, density, elemental weight fraction and thicknesses of the selected spinel ferrites, and setting the physical processes of photoelectric effect, Compton (coherent and incoherent) scattering and pair production corresponding to photon energy. The third stage is the measurement of the simulation value by using GM calculator after 10^6^ hits of gamma radiation on the selected biomaterial at a particular thickness.

## 3. Results and discussion

**Fig. 1:**
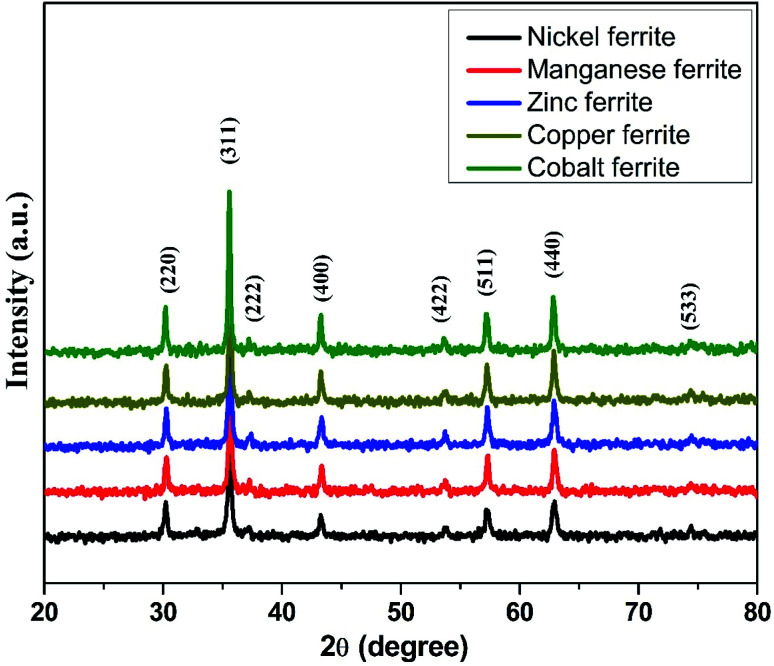
X-ray diffractograms for the produced spinel nano-ferrites.

The room-temperature X-ray diffraction (XRD) outlines of spinel ferrites synthesized *via* the sol–gel auto-ignition technique are presented in Fig. 1. The occurrence of a single intense peak at 2*θ* ∼ 35° is usually related to the most intense (311) diffraction plane characteristics of cubic symmetry. All the reflections observed could be attributed to a cubic spinel lattice, without evidence of additional impurities, showing their single-phase structure. From the XRD profile, the average crystallite sizes of the samples were calculated by the Debye–Scherrer formula based on the diffraction peak of the highly intense (311) plane.^40^ The average crystallite size was found to be in the range of 32–52 nm for all the ferrite samples. The lattice parameter (*a*) obtained by using XRD data was found to lie in the range of 8.3619–8.4742 Å for different spinel ferrites. The structural parameters are shown in Table 1.

**Table 1:** Various structural parameters of nanocrystalline spinel ferrites

Sample	Space group	Average crystallite size (nm) from XRD	Crystallite size (nm) from TEM	Lattice parameter (Å)
NiFe_2_O_4_	*Fd*3̄*m*	36.25	34.02	8.3619
MnFe_2_O_4_	*Fd*3̄*m*	33.87	35.46	8.4742
ZnFe_2_O_4_	*Fd*3̄*m*	32.31	37.62	8.4516
CuFe_2_O_4_	*Fd*3̄*m*	39.75	42.54	8.3839
CoFe_2_O_4_	*Fd*3̄*m*	52.45	56.02	8.3891

**Fig. 2:**
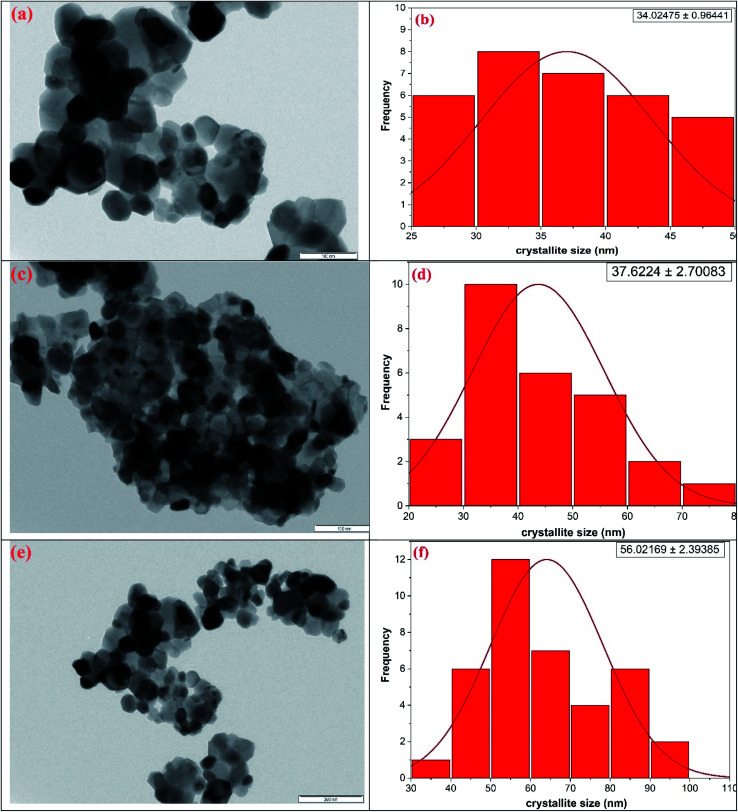
TEM micrographs along with particle size distribution for the representative (a and b) nickel ferrite (c and d) zinc ferrite and (e and f) cobalt ferrite nanocrystals.

The microstructural characteristics of the ferrite nanocrystals were exposed by employing transmission electron microscopy (TEM) as shown in Fig. 2(a–f). Fig. 2(a), (c) and (e) depicts the bright field TEM pictures of the representative nickel ferrite, zinc ferrite and cobalt ferrite nanocrystalline samples, respectively, while the adjacent plots, Fig. 2(b), (d) and (f), demonstrate the crystallite size distribution obtained using the statistical method (Gaussian fitting) from the TEM pictures. Also, the derived mean crystallite sizes are listed in Table 1. The TEM micrographs depict that the surface is composed of homogeneously distributed single and partially polycrystalline facetted grains of varying sizes in nano dimension scale.

We successfully carried out the narrow beam geometry experimental investigations on the manufactured nano-ferrites in pellet form using different gamma ray sources such as ^57^Co (122 keV), ^133^Ba (356 keV), ^22^Na (511 and 1275 keV), ^137^Cs (662 keV), ^54^Mn (835 keV) and ^60^Co (1173 and 1332 keV) and NaI(Tl) detector. The linear attenuation coefficient (*μ*) for the synthesized nickel, manganese, zinc, copper and cobalt nano-ferrites was measured at room temperature for the evaluation of the shielding characteristics with respect to incident photon energy in the range of 122 keV to 1330 keV.

**Fig. 3:**
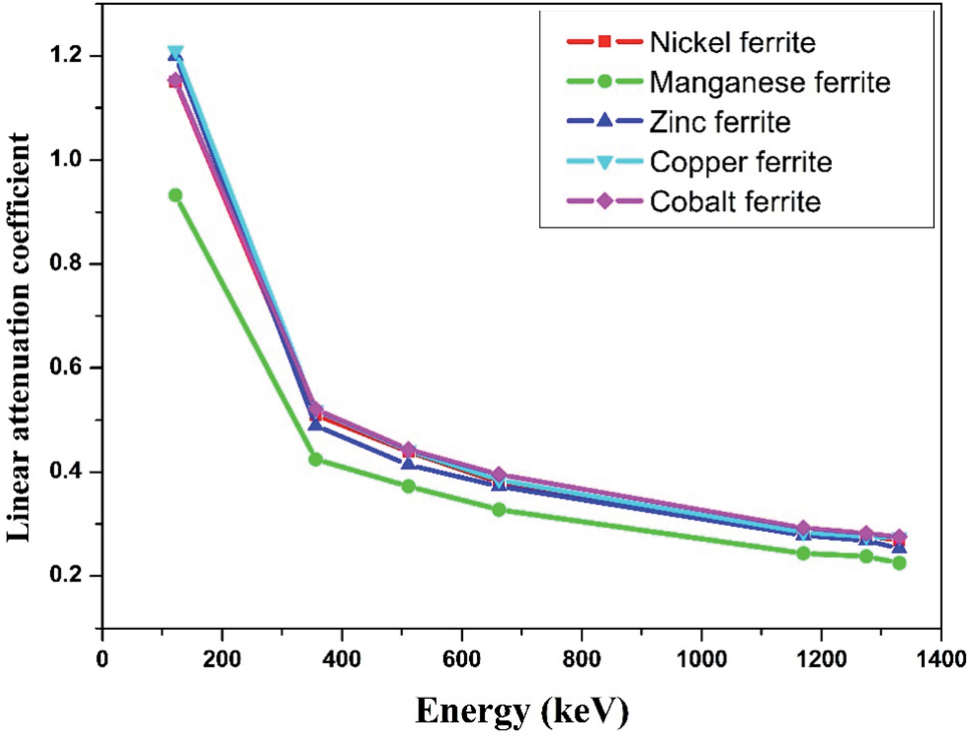
Experimental linear attenuation coefficients (*μ*) *versus* incident photon energy.

The linear attenuation coefficient (*μ*) with respect to photon energy of all the selected nano-ferrites is graphically represented in Fig. 3. It can be observed that as the photon energy increases the linear attenuation coefficient decreases exponentially for all the synthesized nano-ferrites. Zinc and cobalt nano-ferrites exhibit high values of *μ*; however, manganese nano-ferrite displays the lowest value. Also, zinc ferrite with more discrepancy rapidly decreases in the energy range of 122 to 356 keV and then linearly declines with energy compared to the other spinel ferrites. The linear attenuation coefficient is an imperative parameter for the measurement of the shielding characteristics of materials.^41^

**Fig. 4:**
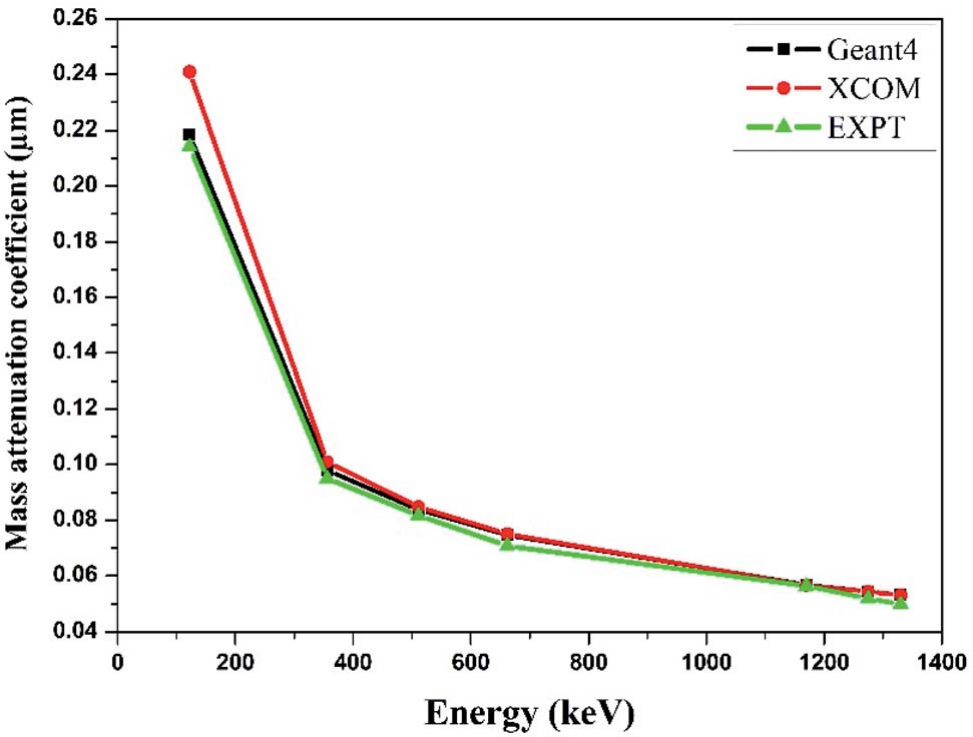
Comparison of experimental and theoretical mass attenuation coefficient against incident photon energy for zinc ferrite.

The mass attenuation coefficient (*μ*_m_) was determined using eqn (1). The theoretical mass attenuation coefficient was simulated by the Monte-Carlo simulation (Geant4) method as well as X-Com (NIST) database as shown in Table 2. The experimental, theoretical and simulated values of the mass attenuation coefficient fairly match with each other for all the synthesized nano-ferrites. Fig. 4 depicts the mass attenuation coefficient (*μ*_m_) *versus* incident photon energy (keV) plot of the representative zinc ferrite sample determined using experimental and theoretical (*viz.* Monto-Carlo simulation and XCOM) techniques, which exhibits the analogous nature. The same trend was observed for all the fabricated nano-ferrites. The value of the mass attenuation coefficient primarily depends on the incident photon energy, and it suddenly decreases with energy (*E* < 400 keV) as photoelectric effect is dominant here; at the moderate energy region the curve remains continual due to Compton scattering phenomenon as reported by Medhat *et al.*^42^ It reflects that Monte-Carlo simulation, experimental and theoretical outcomes are in good agreement with each other. The Monte-Carlo simulation results were more effective than the NIST-XCOM data.

**Table 2:** Mass attenuation coefficient (cm^2^ g^−1^) for the synthesized spinel nano-ferrites

Energy (keV)	Nickel ferrite			Manganese ferrite			Zinc ferrite			Copper ferrite			Cobalt ferrite		
	Geant4	NIST	EXPT	Geant4	NIST	EXPT	Geant4	NIST	EXPT	Geant4	NIST	EXPT	Geant4	NIST	EXPT
122	0.2185	0.2409	0.2142	0.2043	0.2249	0.2023	0.2270	0.2502	0.2308	0.2210	0.2437	0.2241	0.2119	0.2335	0.2086
356	0.0980	0.1010	0.0951	0.0966	0.0993	0.0921	0.0976	0.1006	0.0942	0.0973	0.1001	0.0961	0.0970	0.0998	0.0943
511	0.0839	0.0848	0.0818	0.0828	0.0836	0.0809	0.0832	0.0842	0.0796	0.0830	0.0840	0.0818	0.0830	0.0839	0.0802
662	0.0747	0.0751	0.0708	0.0739	0.0741	0.0711	0.0741	0.0744	0.0717	0.0740	0.0742	0.0712	0.0740	0.0743	0.0715
1170	0.0568	0.0567	0.0564	0.0562	0.0560	0.0528	0.0563	0.0561	0.0535	0.0562	0.0561	0.0526	0.0562	0.0561	0.0529
1275	0.0544	0.0543	0.0519	0.0538	0.0536	0.0516	0.0538	0.0537	0.0516	0.0538	0.0537	0.0511	0.0538	0.0537	0.0510
1330	0.0532	0.0531	0.0499	0.0526	0.0525	0.0488	0.0527	0.0526	0.0486	0.0526	0.0525	0.0507	0.0527	0.0526	0.0498

Nano-magnesium ferrite has greater linear and mass attenuation coefficients compared to that of bulk magnesium ferrite synthesized *via* the standard ceramic technique reported in the literature.^24^ This could be accredited to the homogenous distribution of nano-crystallites with high electron density, which results in higher interaction probability between incident photons and nanocrystals compared to that with bulk ferrite.

Likewise, A. M. El-Khatib *et al.* investigated the gamma attenuation coefficients of micro-sized and nano-sized cadmium oxide (CdO) particles mixed with high-density polyethylene (HDPE) prepared by the compression molding technique.^43^ They observed superior gamma ray shielding characteristics in nano-dimension cadmium oxide compared with that in micro-dimension cadmium oxide.

The effect of particle size on the gamma radiation shielding property of gadolinium oxide dispersed epoxy resin matrix composite was studied by Ran Li *et al.*^44^ They concluded that the produced nano-Gd_2_O_3_ reinforced epoxy composite shows both good shielding and mechanical properties as compared to micro-fillers and suggested this material as a promising novel shielding material for radiation protection purpose.

**Fig. 5:**
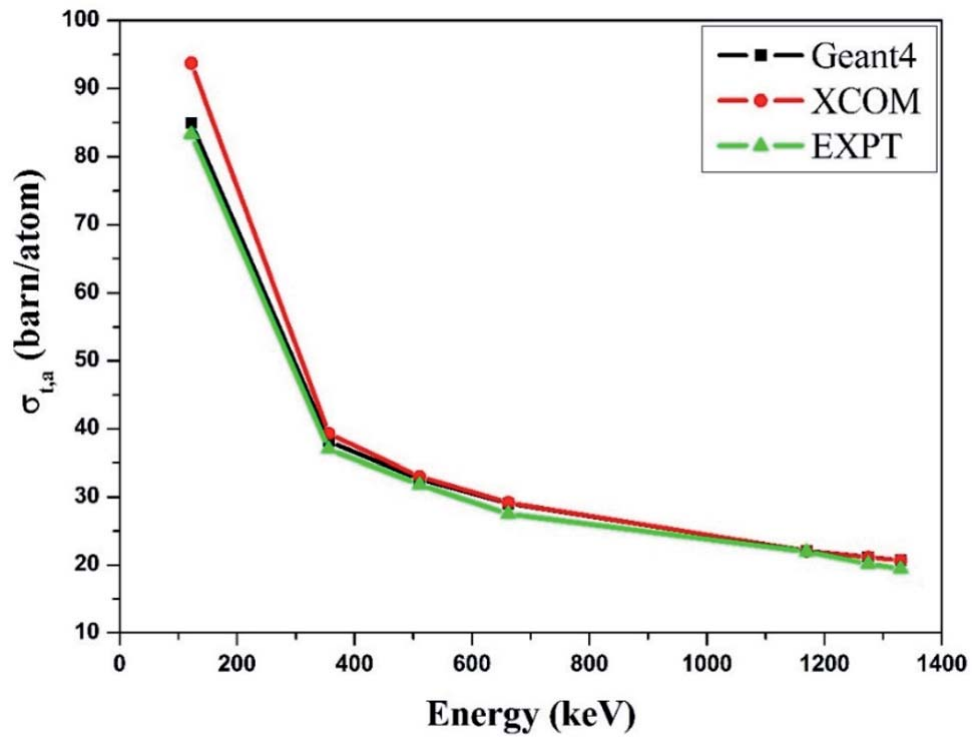
Comparative study of the total atomic cross-section (*σ*_t,a_) verses incident photon energy for zinc ferrite.

**Fig. 6:**
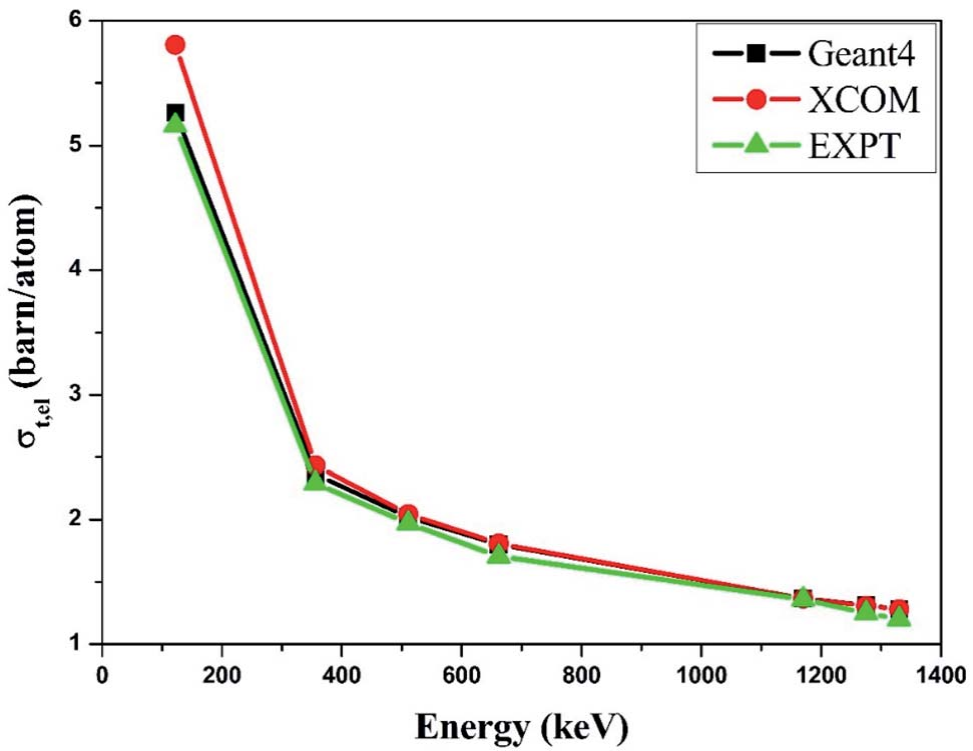
Comparative study of the total electronic cross-section (*σ*_t,el_) *versus* incident photon energy of zinc ferrite.

Similarly, we obtained the total atomic cross section (*σ*_t,at_) and total electronic cross section (*σ*_t,el_) parameters of the nano-ferrites with the help of eqn (4) and (5), respectively. The experimental and theoretical values of *σ*_t,at_ and *σ*_t_,_el_ for all the synthesized nano-ferrites are tabulated in Tables 3 and 4, respectively. The graphical representation of the total atomic cross section (*σ*_t,at_) and total electronic cross section (*σ*_t,el_) of the representative zinc ferrite sample are revealed in Fig. 5 and Fig. 6, respectively. Fig. 5 and 6 show that the experimental results are in good agreement with the Geant4 and XCOM outcomes. Also, they reflect that both *σ*_t,at_ and *σ*_t_,_el_ decrease with increasing incident photon energy.

**Table 3:** Total attenuation cross section *σ*_t,at_ (barn per atom) for the synthesized spinel nano-ferrites

Energy (keV)	Nickel ferrite			Manganese ferrite			Zinc ferrite			Copper ferrite			Cobalt ferrite		
	Geant4	NIST	EXPT	Geant4	NIST	EXPT	Geant4	NIST	EXPT	Geant4	NIST	EXPT	Geant4	NIST	EXPT
122	85.0012	93.7153	83.3284	78.2055	86.0911	77.4399	90.8285	100.1114	92.3490	87.7528	96.7663	88.9837	82.5181	90.9295	81.2330
356	38.1241	39.2912	36.9959	36.9782	37.9926	35.2556	39.0522	40.2526	37.6918	38.6351	39.7469	38.1586	37.7737	38.8524	36.7223
511	32.6389	33.0046	31.8220	31.6956	32.0172	30.9683	33.2904	33.6906	31.8500	32.9569	33.3461	32.4804	32.3218	32.6684	31.2315
662	29.0599	29.1961	27.5427	28.2887	28.3576	27.2169	29.6493	29.7693	28.6890	29.3833	29.4786	28.2715	28.8171	28.9144	27.8435
1170	22.0964	22.0497	21.9408	21.5132	21.4443	20.2117	22.5271	22.4590	21.4067	22.3154	22.2559	20.8860	21.8854	21.8465	20.6003
1275	21.1628	21.1083	20.1902	20.5945	20.5256	19.7523	21.5268	21.4987	20.6465	21.3624	21.3029	20.2904	20.9508	20.9118	19.8604
1330	20.6959	20.6609	19.4122	20.1351	20.0892	18.6805	21.0866	21.0426	19.4461	20.8860	20.8502	20.1315	20.5224	20.4679	19.3931

**Table 4:** Total electronic cross section *σ*_t,el_ (barn per atom) for the synthesized spinel nano-ferrites

Energy (keV)	Nickel ferrite			Manganese ferrite			Zinc ferrite			Copper ferrite			Cobalt ferrite		
	Geant4	NIST	EXPT	Geant4	NIST	EXPT	Geant4	NIST	EXPT	Geant4	NIST	EXPT	Geant4	NIST	EXPT
122	5.2665	5.8064	5.1629	4.8038	5.2882	4.7567	4.7804	5.2690	4.8605	5.5365	6.1051	5.6141	4.8540	5.3488	4.7784
356	2.3621	2.4344	2.2922	2.2714	2.3337	2.1656	2.0554	2.1186	1.9838	2.4375	2.5077	2.4075	2.2220	2.2854	2.1601
511	2.0222	2.0449	1.9716	1.9469	1.9667	1.9022	1.7521	1.7732	1.6763	2.0793	2.1039	2.0492	1.9013	1.9217	1.8371
662	1.8005	1.8089	1.7065	1.7376	1.7419	1.6718	1.5605	1.5668	1.5099	1.8538	1.8598	1.7837	1.6951	1.7008	1.6379
1170	1.3690	1.3662	1.3594	1.3215	1.3172	1.2415	1.1856	1.1821	1.1267	1.4079	1.4042	1.3177	1.2874	1.2851	1.2118
1275	1.3112	1.3078	1.2509	1.2650	1.2608	1.2133	1.1330	1.1315	1.0867	1.3478	1.3440	1.2801	1.2324	1.2301	1.1683
1330	1.2823	1.2801	1.2027	1.2368	1.2340	1.1475	1.1098	1.1075	1.0235	1.3177	1.3155	1.2701	1.2072	1.2040	1.1408

**Fig. 7:**
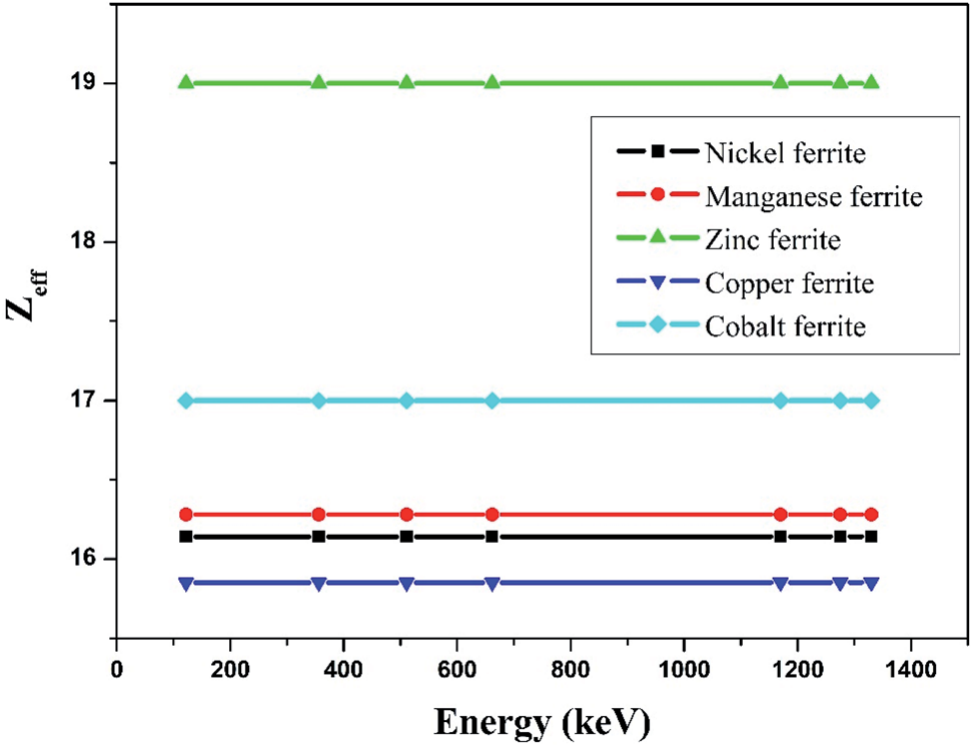
Experimental study of effective atomic number (*Z*_eff_) against incident photon energy.

**Fig. 8:**
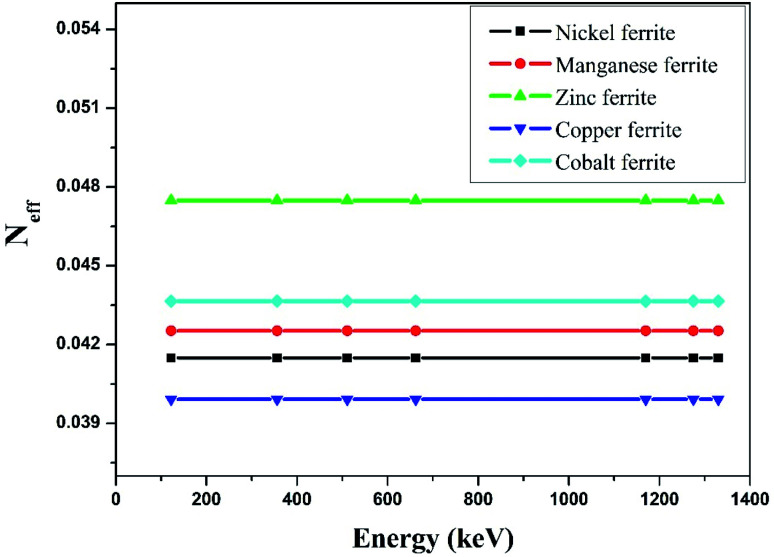
Experimental study of effective electron density (*N*_eff_) against incident photon energy.

The effective atomic number (*Z*_eff_) was estimated by using eqn (6). The value of *Z*_eff_ is graphically represented in Fig. 7 for all the synthesized nano-ferrites against photon energy. It can be observed in Fig. 7 that the value of *Z*_eff_ is declining with rise in photon energy (122–1330 keV). The decreasing behavior of *Z*_eff_ with increasing energy is mainly because the photons live longer due to the photoelectric effect, Compton effect and pair production phenomenon.^45^ The nano-ferrites absorb more photons at higher energy and absorb minimum at lower energy. It reflects that at higher energy the Compton process is dominant, therefore, *Z*_eff_ has a lower value due to the recoiling of the photons (∼1330 keV). Zinc ferrite has the highest *Z*_eff_ value compared with the other synthesized nano-ferrites, and manganese ferrite exhibits the lowest *Z*_eff_ value. Fig. 8 demonstrates the dependence of effective electron density (*N*_eff_) on incident photon energy. *N*_eff_ shows the same behavior as *Z*_eff_ in the incident photon energy range of 122–1330 keV.

The half value layer (HVL), mean free path (mfp) and tenth value layer (TVL) are also vital parameters in predicting the gamma radiation shielding characteristics of the materials. In the present study, we have estimated the half value layer value utilizing relation (8).

**Fig. 9:**
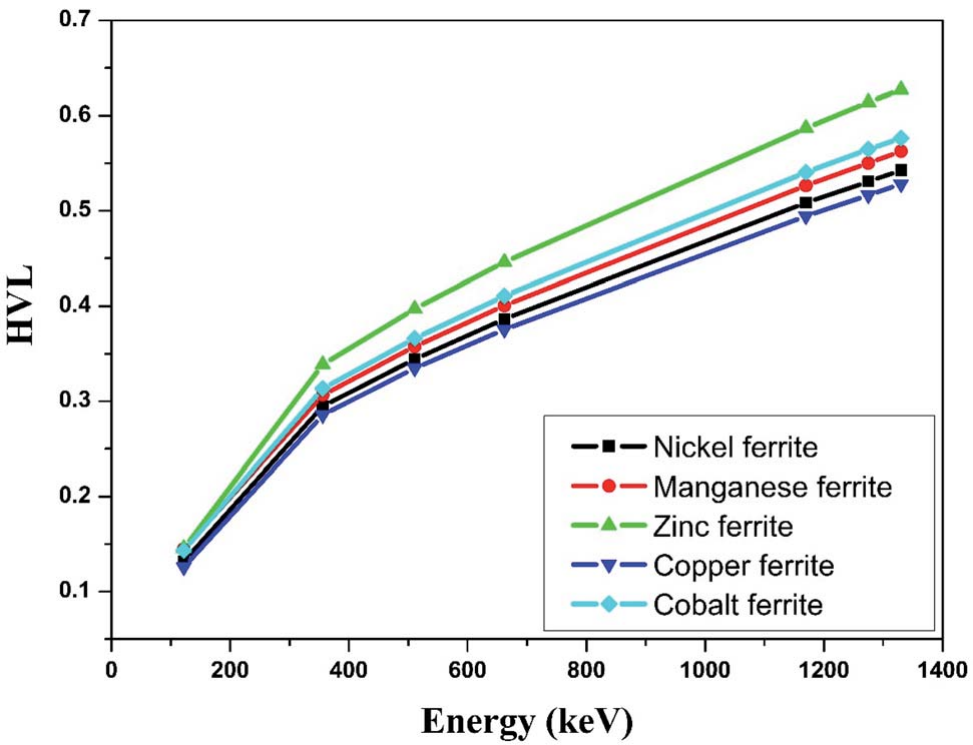
Experimental study of half value layer against incident photon energy.

Table 5 shows the half value layer parameter evaluated using Monte-Carlo simulation, XCOM and experimental data for the synthesized nano-ferrites. Fig. 9 shows the plots of HVL against incident photon energy for all the nano-ferrites. As the incident photon energy increases the half value layer value improves significantly. The zinc ferrite sample exhibits superior HVL values as compared to the other fabricated nano-ferrites. The high HVL value of the synthesized nano-ferrites reflects the gamma ray shielding characteristics.

**Table 5:** Half value layer for the synthesized spinel nano-ferrites

Energy (keV)	Nickel ferrite			Manganese ferrite			Zinc ferrite			Copper ferrite			Cobalt ferrite		
	Geant4	NIST	EXPT	Geant4	NIST	EXPT	Geant4	NIST	EXPT	Geant4	NIST	EXPT	Geant4	NIST	EXPT
122	0.1322	0.1314	0.1348	0.1449	0.1441	0.1463	0.1456	0.1450	0.1432	0.1257	0.1250	0.1240	0.1434	0.1426	0.1457
356	0.2947	0.2938	0.3036	0.3064	0.3060	0.3214	0.3386	0.3382	0.3508	0.2855	0.2853	0.2891	0.3132	0.3128	0.3222
511	0.3442	0.3458	0.3530	0.3575	0.3592	0.3659	0.3972	0.3992	0.4152	0.3347	0.3363	0.3396	0.3661	0.3678	0.3788
662	0.3866	0.3886	0.4079	0.4005	0.4036	0.4163	0.4460	0.4493	0.4609	0.3754	0.3784	0.3902	0.4106	0.4136	0.4249
1170	0.5084	0.5120	0.5490	0.5267	0.5307	0.5606	0.5870	0.5917	0.6178	0.4943	0.4981	0.5282	0.5406	0.5440	0.5744
1275	0.5308	0.5347	0.5564	0.5502	0.5541	0.5736	0.6143	0.6178	0.6405	0.5164	0.5200	0.5437	0.5648	0.5680	0.5958
1330	0.5428	0.5459	0.5787	0.5627	0.5660	0.6066	0.6271	0.6310	0.6800	0.5282	0.5311	0.5480	0.5765	0.5802	0.6101

**Fig. 10:**
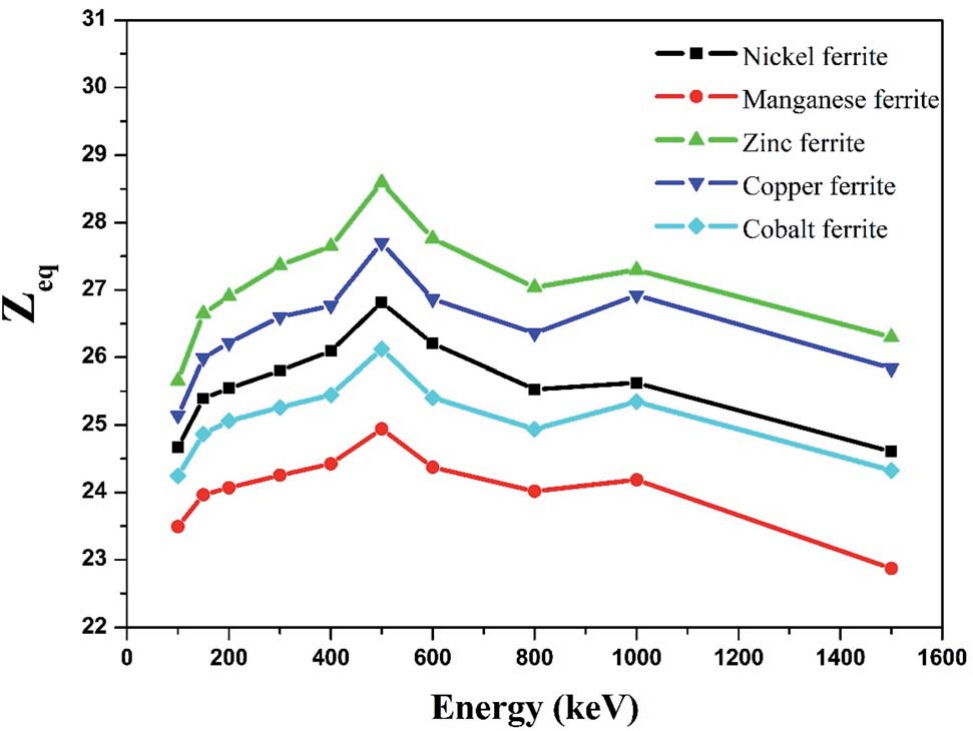
Experimental study of equivalence atomic number (*Z*_eq_) against incident photon energy.

The equivalence atomic number (*Z*_eq_) of nano-ferrites at different photon energies was estimated using eqn (9) and is graphically presented in Fig. 10. The *Z*_eq_ value strongly relies on the incident photon energy. The higher values of *Z*_eq_ at the middle energy range are attributed to the Compton phenomenon, after that the lower or decreasing values of *Z*_eq_ are attributed to the photoelectric process. The *Z*_eq_ is highest for zinc ferrite and lowest for manganese ferrite.

**Fig. 11:**
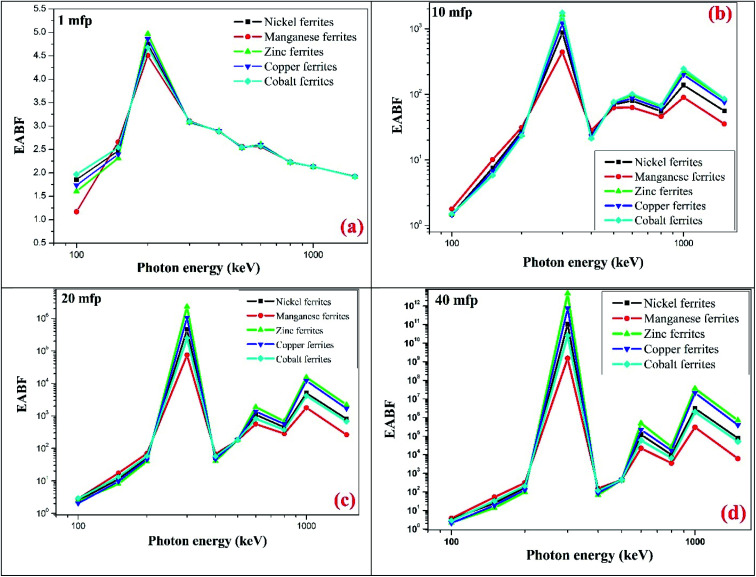
Theoretical EABF values against incident photon energy up to penetration depth (a) 1 mfp (b) 10 mfp (c) 20 mfp and (d) 40 mfp of the synthesized ferrites at photon energy of 100 to 1500 keV.

The variation in the energy absorption buildup factor (EABF) at an incident photon energy of 100–1500 keV up to the penetration depth 1 mfp, 10 mfp, 20 mfp, 40 mfp was estimated using the G-P fitting method. The geometric progression parameter *a*, *b*, *c*, *d* and *X*_k_ of spinel ferrites were determined by using ANSI/ANS-6.4.3-1991 data sheet and the interpolation formula developed by Harima.^46,47^ With the help of Harima's interpolation formulae and ANSI 6.4.3 we also measured the EABF for all the nano-ferrites. Here, *b* is the buildup factor of nano-ferrites and *k* is a multiplication factor for dose at different penetration depths (when *k* ≠ 1). Fig. 11(a–d) shows the variation in the EABF at different penetration depths *viz.* 1 mfp, 10 mfp, 20 mfp and 40 mfp for all the produced spinel ferrite samples. All the spinel ferrites show unique behavior at different energy ranges. The curves of Fig. 11(a–d) reflect that, initially at the low energy region, the EABF increases with increasing photon energy, which is attributed to the dominance of the photoelectric effect, as the photoelectric process is directly proportional to the square of the atomic number and inversely to the cube of its energy.^48^

The EABF enhances significantly in the intermediate region of frequency which can be attributed to Compton process mainly because of multiple scattering of photons. Also, it is observed that the buildup factor value abruptly upsurges in the intermediate region for all penetration depths because of the generation of secondary gamma photons, which demonstrates multiple scattering.^49^ In the third region, as photon energy increases the EABF factor exhibits a simultaneous decreasing and increasing trend, ∼1000 keV, which can be mainly accredited to the pair production phenomenon.

Fig. 11(a–d) clearly depicts that the energy absorption buildup factor increases with increase in penetration depth from 1 mfp to 40 mfp. Zinc ferrite exhibits the highest EABF value and manganese ferrite has the lowest EABF value at the penetration depth of 1 mfp to 40 mfp with photon energy of 100–1500 keV. In earlier reports, B. Singh *et al.* have examined the EABF value of low *Z*_eq_ shielding materials and have observed that the materials with low *Z*_eq_ exhibit a high EABF value.^50^ However, our result shows a greater value of the EABF with higher value of *Z*_eq_. Zinc nano-ferrite exhibits a higher EABF compared with the other spinel ferrite materials.

**Fig. 12:**
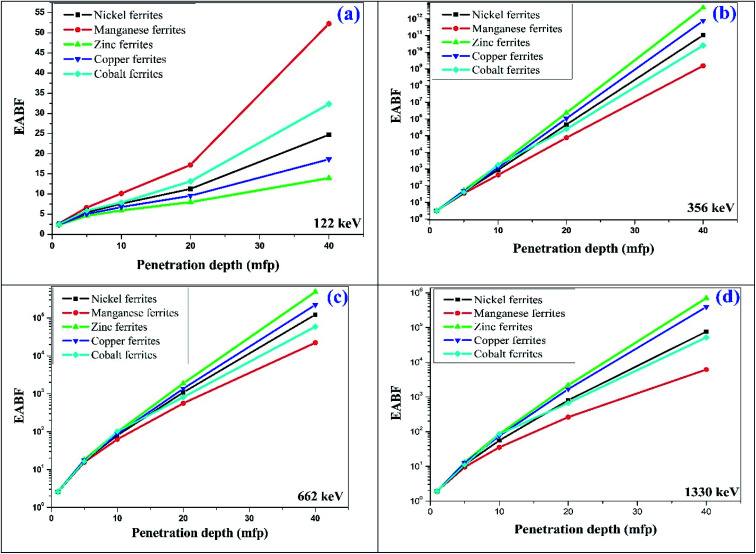
Typical plot of penetration depth against EABF at (a) 122 keV, (b) 356 keV, (c) 662 keV and (d) 1330 keV photon energy for the synthesized spinel ferrites.

The observed values of *Z*_eq_ and EABF reflect that the EABF is dependent on the chemical composition of the materials. For better elucidation of the energy absorption buildup factor with different penetration depths of 1 mfp, 5 mfp, 10 mfp, 20 mfp, 30 mfp and 40 mfp at various photon energies, the EABF as a function of incident photon energy at 122 keV, 356 keV, 662 keV, and 1530 keV is portrayed in Fig. 12(a–d). All the ferrite materials show the same trend, the EABF increases with increasing photon energy. The energy absorption buildup factor is the lowest at 122 keV energy and highest at 356 keV.

Therefore, it can be concluded that nano-spinel ferrite materials are promising highly efficient shielding materials that can be used to reduce radiation dose. The EABF is an important parameter for the measurement of the radiation shielding properties of materials. The obtained EABF values of the present nano-ferrite samples were compared with those of the existing materials utilized for gamma radiation shielding, such as oxide glasses, bismuth borate glasses and marble (for typical 10 MFP). The current samples under investigation show nearly the same order of EABF values at lower photon energy. However, at higher incident photon energy the spinel ferrite nanocrystals show superior values compared with oxide glasses, bismuth borate glasses and marble materials.^51–53^

Also, zinc ferrite displays higher shieling parameter (such as linear attenuation coefficient, effective atomic number, and EABF) values as compared to the other spinel ferrites. This can be understood on the basis of the crystallite size effect; zinc ferrite possesses a lower crystallite size (∼32.31 nm) compared to nickel ferrite (∼36.25 nm), manganese ferrite (∼33.87) copper ferrite (∼39.75 nm) and cobalt ferrite (∼52.45 nm). The obtained spinel ferrites in nano-dimensions are efficient in attenuating radiation since nanomaterials are more uniform and have less agglomeration in the matrix. Also, it is due to the fact that the cross section of photon interaction with material depends on the surface to volume ratio of nanoparticles. Hence, these materials can enhance the shielding ability of the electronic device.

This study reflects the high values of the energy absorption buildup factor for the fabricated spinel ferrite nanomaterials, which can be used for gamma ray shielding purposes, and the performance of electronic components based on spinel ferrite materials in gamma radiation atmosphere.

## 4. Conclusions

In summary, spinel ferrite nanocrystals with high purity were effectively produced by the sol–gel auto-ignition route. Single-phase spinel ferrite nanocrystals of the cubic spinel (*Fd*3̄*m*) phase were confirmed through X-ray analysis. The TEM outcomes showed the finely distributed uniform crystals in nano-dimensions. The mass attenuation coefficient of the ferrites was estimated at distinct incident photon energy in the range of 122–1330 keV. The mass attenuation coefficient, total atomic cross section, total electronic cross section and half value layer determined using Monto-Carlo simulation using Geant4, XCOM (NIST) and experimental procedure were in good agreement. The energy absorption buildup factor was measured at incident photon energy of 100–1500 keV at penetration depths from 1 to 40 mfp *via* the geometric progression (G-P) fitting method. The EABF values were mainly dependent on the chemical composition and *Z*_eq_ of the material. Zinc ferrite displayed a higher EABF value and manganese ferrite had the lowest EABF value. Monte-Carlo simulation *via* Geant4 was found to be the most effective tool for theoretical parameter determination. Zinc ferrite displayed higher shieling parameters as compared to the other spinel ferrites. This can be understood on the basis of the crystallite size effect; zinc ferrite possesses a lower crystallite size (∼32.31 nm) compared to the other produced ferrites. This can be attributed to the homogenous distribution of nano-crystallites with high electron density, which results in higher interaction probability between incident photons and nanocrystals. This experimental and theoretical investigation suggests a new approach towards spinel ferrite nanomaterial at different penetration depths and different energy atmospheres. This gives vital information about the physico-chemical characteristics of spinel ferrites and their shielding properties upon interaction with gamma radiation, which can be applicable in the biomedical field, radiation protection for shielding purposes and performance of electronic appliances in radiation atmosphere.

## Conflicts of interest

There are no conflicts to declare.

## Supplementary Material
